# Supplementary material for: The nucleolar shell provides anchoring sites for DNA untwisting
Source: Commun Biol. 2024 Jan 23;7:83. doi: 10.1038/s42003-023-05750-w (PMC10805735; doi:10.1038/s42003-023-05750-w)
Supplement: Supplementary file 2 — Supplementary Material [file 42003_2023_5750_MOESM2_ESM.pdf]

Supplementary Material

Title: The nucleolar shell provides anchoring sites for DNA untwisting

Authors: Jumpei Fukute[1,2], Koichiro Maki[1,2,3,4]*, Taiji Adachi[1,2,3,4]

[1]Laboratory of Cellular and Molecular Biomechanics, Department of Mammalian Regulatory Network, Graduate School of Biostudies, Kyoto University; 53 Shogoin-Kawahara, Sakyo, Kyoto 606-8507, Japan.

[2]Laboratory of Biomechanics, Institute for Life and Medical Sciences, Kyoto University; 53 Shogoin-Kawahara, Sakyo, Kyoto 606-8507, Japan.

[3]Department of Micro Engineering, Graduate School of Engineering, Kyoto University; 53 Shogoin-Kawahara, Sakyo, Kyoto 606-8507, Japan.

[4]Department of Medicine and Medical Science, Graduate School of Medicine, Kyoto University; 53 Shogoin-Kawahara, Sakyo, Kyoto 606-8507, Japan.

*Corresponding author. Email: maki@infront.kyoto-u.ac.jp

This Supplementary Material includes:

Supplementary Figures 1 to 5

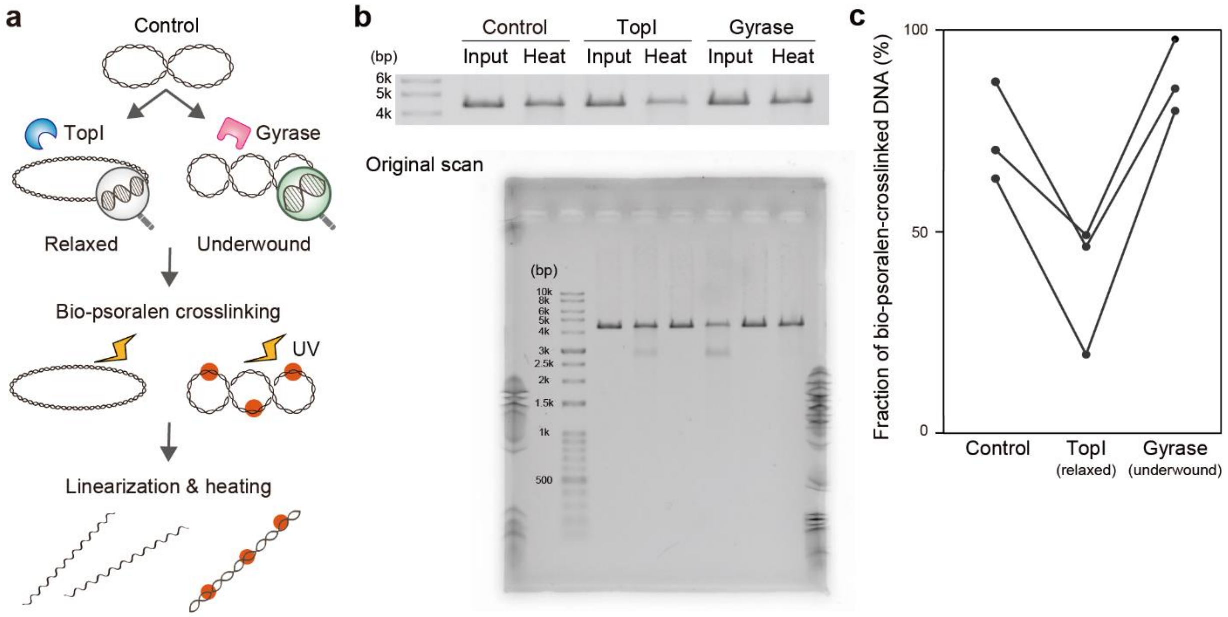

Supplementary Figure 1. *In vitro* biotinylated (bio-)psoralen crosslinking assay. (**a**) Procedure of the *in vitro* crosslinking assay. Relaxed and underwound plasmids (4.2 kb) were prepared by incubating with topoisomerase I (TopI) and gyrase, respectively. Each sample was crosslinked with bio-psoralen under UV irradiation, and linearized by enzymatic treatment. While one half of each sample was directly loaded into the gel (input), the other half was denatured by heat and loaded into the gel (heat). When the sample is heated, un-crosslinked DNA denatured, whereas bio-psoralen-crosslinked DNA retained the double stranded structure. (**b**) Agarose gel electrophoresis of input and heated sample. The bands (4.2 kb) in heated samples correspond to bio-psoralen-crosslinked DNA. (**c**) Fraction (%) of bio-psoralen-crosslinked DNA calculated by dividing the intensity of heated sample by that of the input sample. Three independent biological replicate experiments were performed (n = 3). Data points obtained with the same replicate are connected with a line.

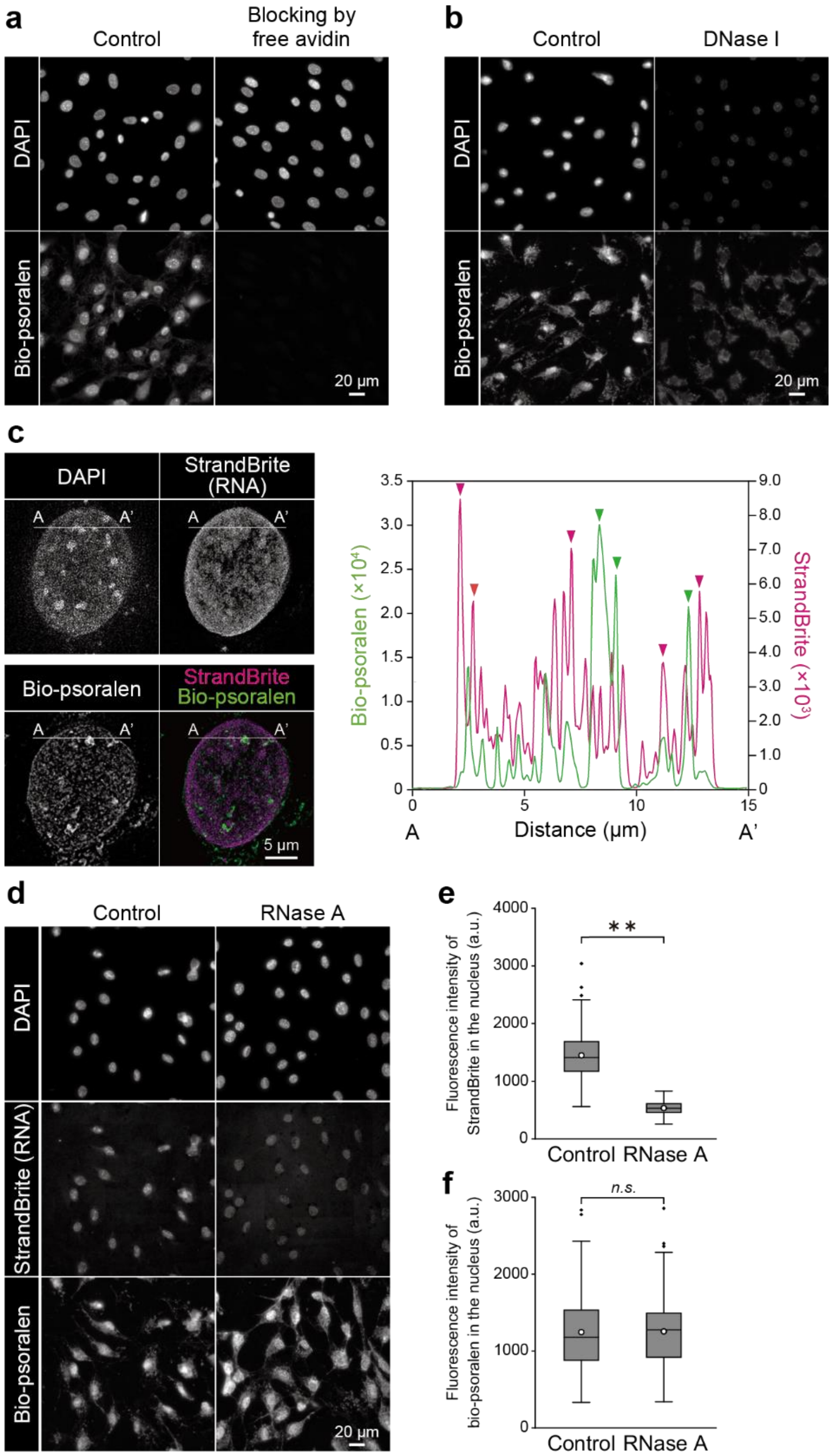

Supplementary Figure 2. Verification of the specificity of bio-psoralen fluorescence imaging for underwound DNA. (**a**) Fluorescence images of 4′,6-diamidino-2-phenylindole (DAPI) and bio-psoralen in samples treated or not with free avidin to block biotin–avidin interaction. (**b**) Fluorescence images of DAPI and bio-psoralen in samples treated or not with DNase I (50 U/mL, 1 h). (**c**) Intranuclear distribution of bio-psoralen and RNA stained with StrandBrite™. (**d**) Fluorescence images of DAPI, RNA, and bio-psoralen in samples treated or not with RNase A (1

mg/mL, 1h). (**e**) Fluorescence intensity of RNA (StrandBrite) in samples treated or not with RNase A (*n* = 150 cells). Statistical significance was assessed using the two-sided Mann–Whitney U test. $P^{**} < 0.01$ ($P = 5.53 \times 10^{-47}$). (**f**) Fluorescence intensity of bio-psoralen in samples treated or not with RNase A (*n* = 150 cells). Statistical significance was assessed using the two-sided Mann–Whitney U test. *n.s.*, not significant ($P = 0.38$)

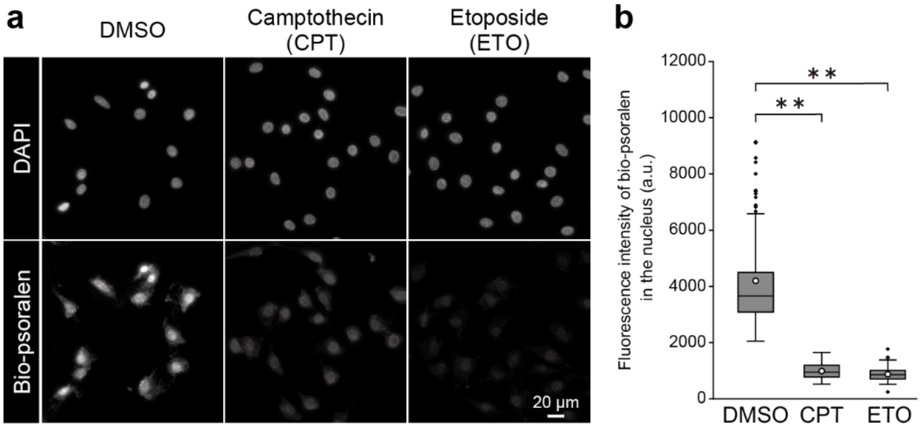

Supplementary Figure 3. Effects of TopI/II inhibition of the fluorescence intensity of bio-psoralen in nuclei. (**a**) Co-staining of DNA (DAPI), bio-psoralen, and NPM in DMSO-, 10 µM Camptothecin-, and 10 µM Etoposide-treated cells. The treatment time is 15 min. Camptothecin and Etoposide inhibit the TOPI and TOPII activities, respectively. (**b**) Fluorescence intensity of bio-psoralen (*n* = 100 cells for each condition). Statistical significance was assessed using the two-sided Steel-Dwass test. $P^{**} < 0.01$ ($P = 0.001$ both for DMSO vs CPT and DMSO vs ETO).

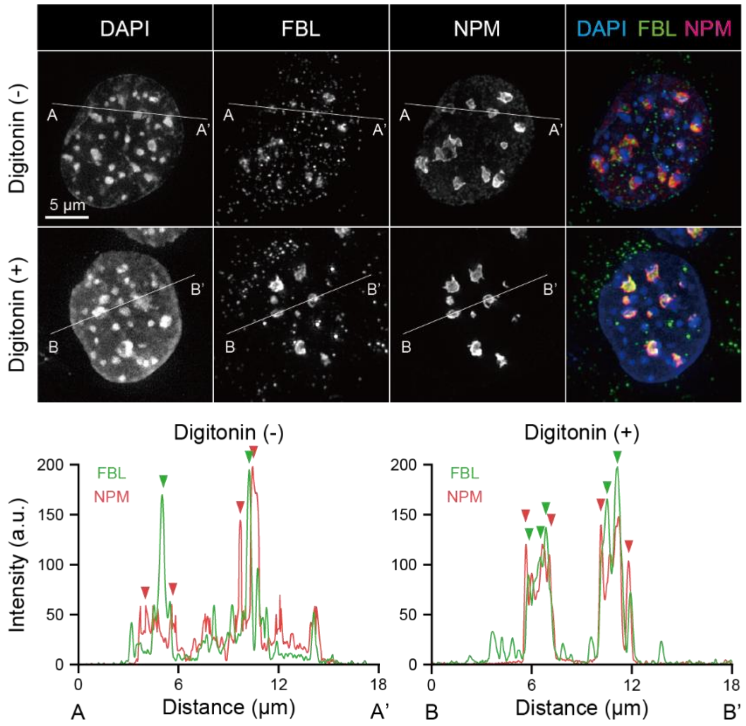

Supplementary Figure 4. Immunofluorescence staining of nucleolar component proteins after digitonin treatment. Co-staining of DNA (DAPI), FBL, and NPM without (top) and with (bottom) digitonin treatment (50 µg/mL, 1 min).

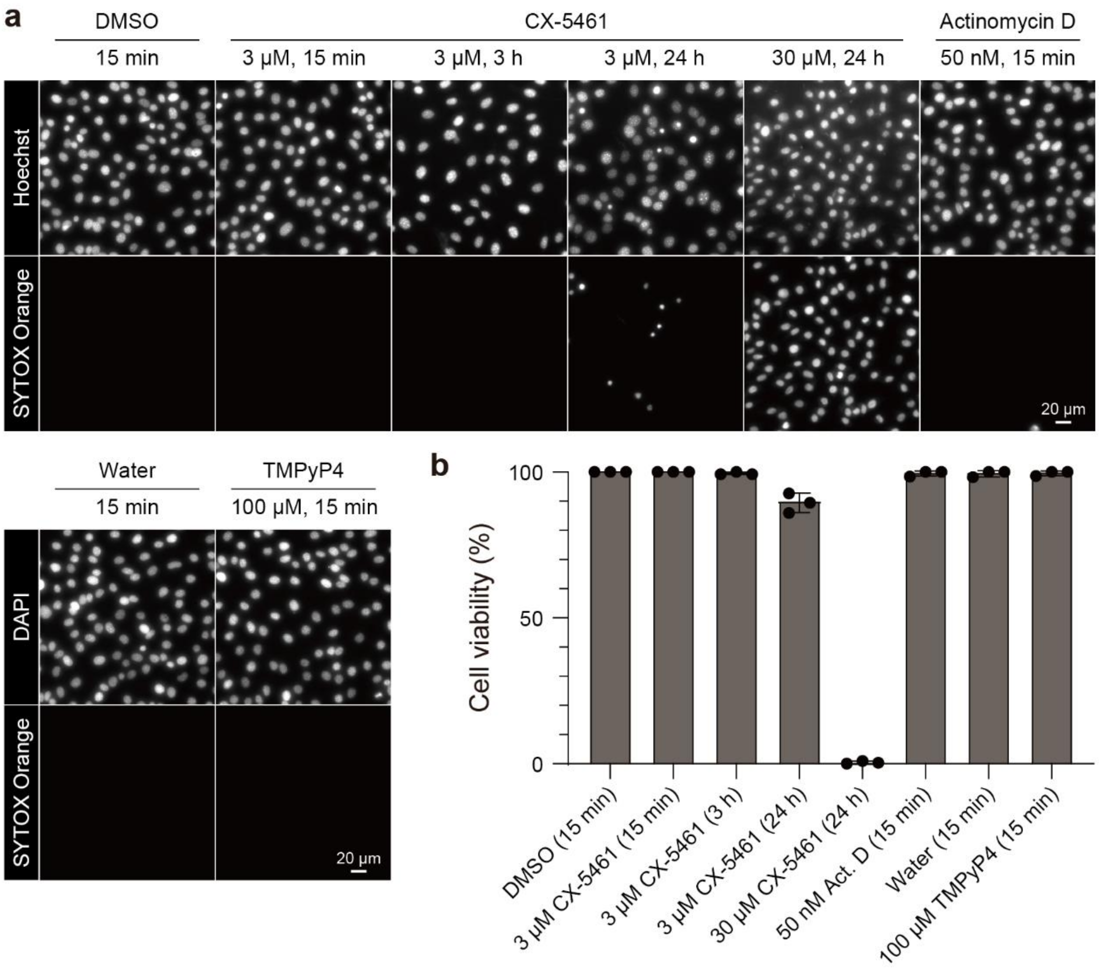

Supplementary Figure 5. Viability of cells treated with CX-5461 and Actinomycin D. (**a**) Dead cell staining for cells treated with DMSO, CX-5461, Actinomycin D, water, and TMPyP4. All cells were stained with Hoechst and dead cells were stained with SYTOX™ Orange. (**b**) Percentage of cell viability (*n* > 45 cells for each condition).
