# Supplementary material for: The nucleolar shell provides anchoring sites for DNA untwisting
Source: Commun Biol. 2024 Jan 23;7:83. doi: 10.1038/s42003-023-05750-w (PMC10805735; doi:10.1038/s42003-023-05750-w)
Supplement: Supplementary file 5 — Reporting Summary [file 42003_2023_5750_MOESM5_ESM.pdf]

nature portfolio

Corresponding author(s): Koichiro Maki, COMMSBIO-23-3021-A

Last updated by author(s): Dec 20, 2023

# Reporting Summary

Nature Portfolio wishes to improve the reproducibility of the work that we publish. This form provides structure for consistency and transparency in reporting. For further information on Nature Portfolio policies, see our Editorial Policies and the Editorial Policy Checklist.

## Statistics

For all statistical analyses, confirm that the following items are present in the figure legend, table legend, main text, or Methods section.

| n/a | Confirmed | |
|---|---|---|
| ☐ | ☒ | The exact sample size (*n*) for each experimental group/condition, given as a discrete number and unit of measurement |
| ☐ | ☒ | A statement on whether measurements were taken from distinct samples or whether the same sample was measured repeatedly |
| ☐ | ☒ | The statistical test(s) used AND whether they are one- or two-sided<br>*Only common tests should be described solely by name; describe more complex techniques in the Methods section.* |
| ☒ | ☐ | A description of all covariates tested |
| ☒ | ☐ | A description of any assumptions or corrections, such as tests of normality and adjustment for multiple comparisons |
| ☐ | ☒ | A full description of the statistical parameters including central tendency (e.g. means) or other basic estimates (e.g. regression coefficient) AND variation (e.g. standard deviation) or associated estimates of uncertainty (e.g. confidence intervals) |
| ☐ | ☒ | For null hypothesis testing, the test statistic (e.g. $F$, $t$, $r$) with confidence intervals, effect sizes, degrees of freedom and $P$ value noted<br>*Give P values as exact values whenever suitable.* |
| ☒ | ☐ | For Bayesian analysis, information on the choice of priors and Markov chain Monte Carlo settings |
| ☒ | ☐ | For hierarchical and complex designs, identification of the appropriate level for tests and full reporting of outcomes |
| ☒ | ☐ | Estimates of effect sizes (e.g. Cohen's *d*, Pearson's *r*), indicating how they were calculated |

*Our web collection on statistics for biologists contains articles on many of the points above.*

## Software and code

Policy information about availability of computer code

| | |
|---|---|
| Data collection | cellSense (version 3.2) was used for capturing epifluorescence images.<br>Zen (version 3.6) was used for structured illumination microscopy imaging. |
| Data analysis | Fiji (Image J, version 1.53t) was used for image analysis.<br>3D Suite (version 4.1.5) was used for 3D image analysis.<br>G4Hunter was used for prediction of putative G4-forming sequences.<br>SciPy (version 1.11.1) was used for statistical analysis. |

For manuscripts utilizing custom algorithms or software that are central to the research but not yet described in published literature, software must be made available to editors and reviewers. We strongly encourage code deposition in a community repository (e.g. GitHub). See the Nature Portfolio guidelines for submitting code & software for further information.

## Data

Policy information about availability of data

All manuscripts must include a data availability statement. This statement should provide the following information, where applicable:

- Accession codes, unique identifiers, or web links for publicly available datasets
- A description of any restrictions on data availability
- For clinical datasets or third party data, please ensure that the statement adheres to our policy

The datasets generated and/or analyzed during the current study are available from the corresponding author on reasonable request.

## Research involving human participants, their data, or biological material

Policy information about studies with human participants or human data. See also policy information about sex, gender (identity/presentation), and sexual orientation and race, ethnicity and racism.

| | |
|---|---|
| Reporting on sex and gender | No human participant/data is included in this study. |
| Reporting on race, ethnicity, or other socially relevant groupings | No human participant/data is included in this study. |
| Population characteristics | No human participant/data is included in this study. |
| Recruitment | No human participant/data is included in this study. |
| Ethics oversight | No human participant/data is included in this study. |

Note that full information on the approval of the study protocol must also be provided in the manuscript.

# Field-specific reporting

Please select the one below that is the best fit for your research. If you are not sure, read the appropriate sections before making your selection.

☒ Life sciences ☐ Behavioural & social sciences ☐ Ecological, evolutionary & environmental sciences

For a reference copy of the document with all sections, see nature.com/documents/nr-reporting-summary-flat.pdf

# Life sciences study design

All studies must disclose on these points even when the disclosure is negative.

| | |
|---|---|
| Sample size | No statistical methods were used to predetermine the sample sizes. |
| Data exclusions | No data was excluded. |
| Replication | We performed biological replicates at minimum of 3 times. |
| Randomization | Our study design did not require randomization. |
| Blinding | Our study design did not require blinding. |

# Reporting for specific materials, systems and methods

We require information from authors about some types of materials, experimental systems and methods used in many studies. Here, indicate whether each material, system or method listed is relevant to your study. If you are not sure if a list item applies to your research, read the appropriate section before selecting a response.

## Materials & experimental systems

| n/a | Involved in the study |
| --- | --- |
| | ☒ Antibodies |
| | ☒ Eukaryotic cell lines |
| ☒ | Palaeontology and archaeology |
| ☒ | Animals and other organisms |
| ☒ | Clinical data |
| ☒ | Dual use research of concern |
| ☒ | Plants |

## Methods

| n/a | Involved in the study |
| --- | --- |
| ☒ | ChIP-seq |
| ☒ | Flow cytometry |
| ☒ | MRI-based neuroimaging |

# Antibodies

Antibodies used

For immunofluorescence, following primary antibodies were used.
anti-RNA polymerase II CTD repeat YSPTSPS (phospho S2) (rabbit polyclonal, 5095, Abcam, Lot#: GR3279443-1)
anti-topoisomerase I (rabbit polyclonal, 85038, Abcam, Lot#: GR3374463-1)
anti-RPA194 (mouse monoclonal, 48385, Santa Cruz Biotechnology, Lot#: F1421)
anti-fibrillarin (rabbit monoclonal, 2639, Cell Signaling Technology, Lot#: 4)
anti-NPM 1 (mouse monoclonal, 32-5200, Invitrogen, Lot#: WK338140)

Validation

All primary antibodies are commercially available and validated by the manufactures.
anti-RNA polymerase II CTD repeat YSPTSPS (phospho S2) (5095, Abcam): https://www.abcam.co.jp/products/primary-antibodies/rna-polymerase-ii-ctd-repeat-ysptsps-phospho-s2-antibody-ab5095.html
anti-topoisomerase I (85038, Abcam): https://www.abcam.co.jp/products/primary-antibodies/topoisomerase-i-antibody-ab85038.html
anti-RPA194(48385, Santa Cruz Biotechnology): https://www.scbt.com/ja/p/rpa194-antibody-c-1
anti-fibrillarin(2639, Cell Signaling Technology): https://www.cellsignal.com/products/primary-antibodies/fibrillarin-c13c3-rabbit-mab/2639
anti-NPM 1(32-5200, Invitrogen): https://www.thermofisher.com/antibody/product/NPM1-Antibody-clone-FC-61991-Monoclonal/32-5200

# Eukaryotic cell lines

Policy information about cell lines and Sex and Gender in Research

Cell line source(s)

MC3T3-E1 cells were purchased from RIKEN CELL BANK (https://cell.brc.riken.jp/ja/)

Authentication

None of the cell lines used were authenticated.

Mycoplasma contamination

Cell lines were confirmed mycoplasma free.

Commonly misidentified lines
(See ICLAC register)

No commonly misidentified cell lines were used.
